# Routine clinical chemistry and haematological test reference intervals for healthy adults in the Bhutanese population

**DOI:** 10.1371/journal.pone.0273778

**Published:** 2022-09-01

**Authors:** Kuenzang Dorji, Sonam ChhodenR, Kinley Wangchuk, Sonam Zangpo, Shacha Tenzin, Chenga Dawa, Puja Devi Samal, Nima, Jigme Tshering, Choney Wangmo, Sonam Zangpo, Kinley Dorji, Sonam Tshewang

**Affiliations:** 1 Quality Assurance and Standardization Division, Secretariat, Ministry of Health, Thimphu, Bhutan; 2 Faculty of Postgraduate Medicine, Khesar Gyalpo University of Medical Sciences of Bhutan, Thimphu, Bhutan; 3 Department of Pathology and Laboratory Medicine, Jigme Dorji Wangchuck National Referral Hospital, Thimphu, Bhutan; 4 Department of Pathology and Laboratory Medicine, Eastern Regional Referral Hospital, Mongar, Bhutan; 5 Department of Pathology and Laboratory Medicine, Central Regional Referral Hospital Gelephu, Gelephu, Bhutan; 6 Department of Pathology and Laboratory Medicine, Bajo Hospital, Wangdue Phodrang, Bhutan; 7 Department of emergency, Bajo Hospital, Wangdue Phodrang, Bhutan; Stellenbosch University Faculty of Medicine and Health Sciences, SOUTH AFRICA

## Abstract

Laboratory medicine plays a critical role in the modern healthcare system, and it is reported to influence 60–70% of clinical decision makings. The quantitative laboratory test results are interpreted by comparing to the Reference Intervals (RIs) and therefore the use of appropriate RIs is critical. Clinical laboratories in Bhutan have been randomly using RIs from textbooks and manufacturer’s package inserts without even verifying their applicability and therefore lessening their contribution to clinical decision makings. To improve the healthcare service delivery in Bhutan, this study aims to establish routine clinical chemistry and haematological test RIs for healthy adults in the Bhutanese population. Out of 1150 (male, n = 570; female, n = 580) healthy Bhutanese adults listed for the study through a simple random sampling technique, 1002 (male, n = 405; female, n = 597) individuals were assessed and 815 (male, n = 372; female, n = 443) individuals were enrolled in the study. An adequate volume of venous blood was drawn from these participants with the use of standard phlebotomy technique for clinical chemistry and haematological analysis. The laboratory data were analysed with the use of statistical methods recommended by the International Federation of Clinical Chemistry and Laboratory Medicine and Clinical and Laboratory Standards Institute. After excluding the test results indicating underlying pathology and statistically detected outliers, a maximum of 775 (male, n = 346; female, n = 429) and 784 (male, n = 351; female, n = 433) individuals test values were eligible for clinical chemistry and haematology RIs establishment respectively. Statistically, there were no significant differences between age groups of same-sex for both test categories; however, significant differences between sex were observed for various test parameters in both test categories. Our RIs are generally comparable to other published literature. The established RIs are applicable to all the adult Bhutanese population; however, clinical laboratories should validate the transference of these RIs before using them for clinical purposes.

## Introduction

Laboratory medicine has evolved as an integral part of the modern healthcare system. Laboratory test results play a critical role in clinical decision making by aiding in diagnosis, prognostication, patient management and preventive healthcare [1, 2]. The laboratory test results were reported to influence 60–70% of the clinical decisions thus indicating its cruciality in healthcare service delivery [3, 4]. The comparison is fundamental in any scientific discipline, likewise, quantitative laboratory test results are valuable only when they can be compared to the spread of values found in a health or disease. The Reference Intervals (RIs) are the most important tool used to interpret quantitative laboratory results; therefore, the quality of the RIs is as important as the quality of the test result itself [5–7]. The concept of the RIs was introduced in 1969 and its establishment processes were refined over the period [7, 8]. The concerned international organisations have recommended individual laboratories establish their own RIs based on the methods of analysis used and the population served; however, laboratories can alternatively validate and adopt the published RIs through the standard approach [9, 10]. Despite the clear directives, due to complex scientific procedures and financial difficulties involved, many clinical laboratories particularly in non-industrialised countries prefer not to establish their RIs [11–14]. In line with this trend, the clinical laboratories in Bhutan have been randomly using RIs from textbooks and manufacturer’s package inserts without even verifying their applicability. This potentially results in sub-optimal interpretation of laboratory test results and thus lessening its contribution to clinical decision making.

Bhutan is a landlocked kingdom in the eastern Himalayas sandwiched between China in the north and India in the south, west and east. It has an area of 38,394 km^2^ and a projected population of 727,145 (47.7% female) persons [15, 16]. All the healthcare centres in the country are owned by the government and currently, there are 289 healthcare centres (51 hospitals, 184 primary healthcare centres and 54 sub-post clinics) in the country [17]. The healthcare centres provide a range of clinical laboratory services as per the standard set by the Ministry of Health, Royal Government of Bhutan. Since the laboratory test results largely influence clinical decision makings, the improvement of clinical laboratory services in Bhutan through the establishment of RIs specific to the Bhutanese population can improve the quality of healthcare service delivery in Bhutan. Therefore, as per the International Federation of Clinical Chemistry and Laboratory Medicine (IFCC) and Clinical and Laboratory Standards Institute (CLSI) guidelines [18–22], this study aims to establish routine clinical chemistry and haematological test RIs for healthy adults in the Bhutanese population.

## Materials and methods

### Ethical considerations and selection of reference individuals

The study protocol was approved by the Research Ethics Board for Health (REBH), Ministry of Health, Bhutan. The samples were collected only after obtaining written informed consent from the participants. For illiterate participants, consent was gained by obtaining the participant’s thumbprint and the witness’s signature after reading and explaining the study information sheet and certificate of consent using appropriate language in the presence of a literate witness. As per the REBH requirement, optimal confidentiality was maintained to store all the information (demographic details, clinical details, and laboratory results) of the participants and made accessible only to the authorised persons. The study was carried out over 24 months (01-03-2020 to 28-02-2022).

The stratified random sampling technique was followed in this study. The Kingdom of Bhutan is officially divided into 20 Dzongkhags (districts) which are further divided into Gewogs (block) and Chiwogs (sub-blocks). For this research purpose, these Dzongkhags were classified into four categories based on the predominant ethnic population (Tshangla, Ngalop, Lhotshampa and Highlander). Dzongkhags with a highlander population were re-listed for selection. From each category, the Dzongkhags for sampling were selected based on the simple random sampling technique. Through this sampling process, Mongar, Pemagatshel, Lhuentshe, Trashigang and Trashiyangtshe Dzongkhags were selected for Tshangla ethnic category sampling. The Wangdue Phodrang, Gasa and Punakha Dzongkhags were selected for Ngalop ethnic category sampling. Sarpang, Tsirang and Samdrupjongkhar Dzongkhags were selected for Lhotshampa ethnic category. Similarly, Trashigang (Merak and Sakteng) and Gasa (Laya) Dzongkhags were selected for the highlander ethnic category. Once the Dzongkhags were selected, the healthcare centres in that Dzongkhag were listed and selected for sampling and the public registry of the selected healthcare centre is then used to select the reference individual through the same sampling technique. The participants were screened or examined with the use of pre-defined questionnaires and excluded from the study if they met any of the exclusion criteria (Table 1).

**Table 1:** Exclusion criteria. The participants and blood samples were excluded from the study if they met any of the following criteria.

S.no	Exclusion criteria
1	Abnormal body mass index: ≥30 kg/m2 or <18.5Kg/m2
2	Acute or chronic infection (tuberculosis, malaria etc.)
3	Chronic liver or kidney disease
4	Alcoholism: ≥70g per day (equivalent to 5 alcoholic drinks)
5	Diabetes treated with oral therapy or insulin
6	Drug abuse and treatment for disease or suffering
8	Genetically determined risk
9	Hospitalized or seriously ill in the past 4 weeks
10	Hypertension
11	Known carrier of HBV, HCV, or HIV
12	On oral contraceptives
13	Neoplasms
14	Pregnancy or within one year after childbirth
15	Transfusion (blood or components) in the last 4 months
16	Organ donors and recipients
17	Skin rashes
18	Tobacco usage: greater than 20 pieces per day
19	Blood donation in the last 3 months
20	Breastfeeding
21	Participated in a research study involving investigational drugs in the past 12 weeks
22	Any other significant disease or ailment that an investigator feels may have an impact on the study’s findings.
23	Abnormal blood test results.
24	Specimen nonconformity (haemolysed sample, lipemic sample, clotted sample for whole blood or plasma analysis, inappropriate volume and wrong tubes or additives)

### Pre-analytical and analytical considerations

The participants were asked to continue their routine diet and activities and their samples were collected between 9 am– 5 pm. The participants were comfortably seated in a chair and blood samples were collected from the antecubital fossa with the use of the standard phlebotomy technique. From each participant, 10 ml of venous blood was drawn, and it was transferred to different tubes (K2EDTA, Sodium citrate, sodium fluoride and serum separator tube). The blood samples were gently mixed by inverting 8–10 times for thorough mixing of blood and anticoagulant or to activate clotting. The samples for clinical chemistry and Prothrombin Time (PT) analysis were allowed to sit for 30–40 minutes and then centrifuged at 3500 RPM for 5–10 minutes to obtain the serum or plasma. The samples were transported as soon as possible to the testing centre for analysis in a shipment box maintained at 2–8°C.

The Complete Blood Count (CBC, 24 parameters) analysis was performed with the use of Sysmex XS-1000i or XS-800i (Sysmex, Japan) and reagents from the same manufacturer. The PT testing was performed with the use of the HumaClot Pro fully automated coagulation analyser (Human, Diagnostics Worldwide) and Hemostat Thromboplastin-SI reagent or manually using the same reagent in the absence of coagulation analysers. The clinical chemistry analysis (20 parameters) was carried out with the use of a Mispa Nano Plus fully automatic clinical chemistry analyser (Agappe Diagnostics Limited, India) and reagents from the same manufacturer. All the clinical chemistry analyses were carried out at 37±0.1°C. The electrolyte analysis (3 parameters) was performed with the use of Elite Electrolyte Analyzer (Trivitron Healthcare, India). The principle of analyses of all test parameters is shown in Table 2.

**Table 2:** The sample used and the principle of analysis of test parameters.

S.no	Parameter	Sample	Principle of analysis
**Clinical chemistry**			
1	Alb	Serum	Bromocresol green method
2	TP	Serum	Direct biuret method
3	ALP	Serum	Kinetic method recommended by the IFCC
4	D-Bil	Serum	Diazo method
5	T-Bil	Serum	Modified TAB method
6	SGOT	Serum	Kinetic, IFCC method
7	SGPT	Serum	Kinetic, IFCC method
8	AMY	Serum	CNPG3 method
9	GGT	Serum	Szasz method
10	Ca	Serum	Modified arsenazo III method
11	Chol	Serum	CHOD-PAP method
12	TGL	Serum	GPO-TOPS method
13	Glu-r	Plasma	Hexokinase method
14	CK	Serum	Optimized IFCC method
15	Cr	Serum	Jaffe’s reaction, alkaline picrate method
16	Urea	Serum	Urease/GLDH method
17	LDH	Serum	Scandinavian recommended method
18	Mg	Serum	Xylidyl blue with ATCS method
19	PO4	Serum	Phosphomolybdate method
20	Na	Serum	Indirect ISE method
21	K	Serum	Indirect ISE method
22	Cl	Serum	Indirect ISE method
23	UA	Serum	Uricase-PAP method
**Haematology**			
24	CBC	Whole blood	• Hydro-Dynamic Focusing (DC detection) • Flow cytometry method (using a semiconductor laser) • SLS haemoglobin method
25	PT	Plasma	One-stage PT measurement

The quality of all the laboratory analyses was ensured by running commercial Quality Control (QC) materials before the sample analysis and at pre-defined intervals. The precision and accuracy of CBC analysis were ensured by running e-CHECK (XS) QC materials (three-level) manufactured by Sysmex, Japan. Furthermore, the automated CBC results were correlated with blood smear findings at regular intervals. The quality of PT analysis was controlled with the use of Hemostat Control Plasma (two-level) manufactured by the Human, Germany. The quality of Clinical chemistry analysis was ensured by running a Qualicheck (two-level) QC material manufactured by Agappe, India.

### Data analysis and reference intervals

The data were analysed with the use of Minitab 17 (17.1.0) statistical software package and Microsoft Excel (Microsoft 365 MSO, Version 2111 Build 16.0.14701.20206). All the statistical analyses were performed at the significance level of 0.05. The data was primarily partitioned based on sex and furthermore classified into three sub-categories based on age (18–31, 32–45 and 46–60 years). The frequency histogram of all the parameters in each sex was visually inspected to get the general idea of the data distributions and outlier(s) presence. The outlier(s) were confirmed and excluded with the use of Dixon–Reed, Tukey, and Grubb’s tests. For each test parameter, a One-Way Analysis of Variance (ANOVA) was performed between the sex and age group categories under each sex to determine whether there are significant differences among the means of the populations. Simultaneously, the means were compared for the difference using Tukey’s Pairwise Comparisons. The Anderson-Darling normality test was used for data normality testing. The RI for data with a Gaussian distribution is estimated with the use of a Gaussian percentile estimate ($\bar{x}$±1.96SD) and for non-Gaussian, the RI is estimated with the use of a non-parametric percentile estimate (2.5^th^ and 97.5^th^ values). In the non-parametric RI estimate, if the rank is not an integer, the precise limits were obtained by interpolating the two closest observations. Both RI estimation covers the central 95% of the reference values. The 90% confidence interval of the RI is calculated as per the CLSI and IFCC guidelines.

## Results

### Demographic profile

Out of 1150 (male, n = 570; female, n = 580), healthy Bhutanese adults listed as the reference individual, 1002 (87.13%; male, n = 405; female, n = 597) individuals turned up to participate in the study. After screening this population with the use of pre-set questionnaires, 815 (81.34%; male, n = 372; female, n = 443) individuals were selected for the study. After excluding laboratory results indicating underlying pathology and statistically detected outliers for each test parameter, the maximum of 775 (male, n = 346; female, n = 429) individuals’ clinical chemistry test values were eligible for RI establishment. Among the males, 32.95%, 37.00% and 30.06% of the individuals were within the age range of 18–31, 32–45 and 46–60 years respectively. Similarly in females, 36.83%, 39.16% and 24.01% of the individuals were within the age range of 18–31, 32–45 and 46–60 years respectively. The ethnic distribution, education level and occupation of the clinical chemistry reference sample group are shown in Table 3.

**Table 3:** The demographic details (ethnic distribution, education level and occupation) of the clinical chemistry and haematology reference sample group.

Characteristic	Male	Female
	**Clinical chemistry**	
**Ethnic**		
Tshangla	112 (32.37%)	138 (32.17%)
Ngalop	102 (29.48%)	121(28.21%)
Lhotshampa	100 (28.90%)	111 (25.87%)
Highlander	32 (9.25%)	59 (13.75%)
	**Haematology**	
**Ethnic**		
Tshangla	109 (31.05%)	141 (32.56%)
Ngalop	102 (29.06%)	130 (30.02%)
Lhotshampa	106 (30.20%)	100 (23.09%)
Highlander	34 (9.69%)	62 (14.32%)
	**Clinical chemistry and haematology**	
**Occupation**		
Farmers	173 (49.29%)	261 (60.28%)
Civil servant	53 (15.10%)	39 (9.01%)
Corporate employee	10 (2.85%)	12 (2.77%)
Business personnel	35 (9.97%)	36 (8.31%)
Students	46 (13.11%)	33 (7.62%)
Religious personnel	14 (3.99%)	5 (1.15%)
others	20 (5.70%)	47 (10.85%)
	**Clinical chemistry and haematology**	
**Education level**		
Primary education	65 (18.52%)	57 (13.16%)
High school	104 (29.63%)	101 (23.33%)
Bachelor’s degree or higher	22 (6.27%)	21 (4.85%)
Others	52 (14.81%)	74 (17.09%)
Illiterate	108 (30.77%)	180 (41.57%)

For haematology, a maximum of 784 (male, n = 351; female, n = 433) individuals’ test values were eligible for RI establishment. Among the males, 33.62%, 34.76% and 31.62% of the individuals were within the age range of 18–31, 32–45 and 46–60 years respectively. Similarly in females, 36.03%, 40.65% and 23.33% of the individuals were within the age range of 18–31, 32–45 and 46–60 years respectively. The ethnic distribution, education level and occupation of the haematology reference sample group are shown in Table 3.

### Clinical chemistry RIs

The age range of individuals constituting the clinical chemistry reference sample group for both sexes was 18–60 years old. Within the same sex, there was no significant difference between the age group categories for all test parameters (Table 4).

**Table 4:** The statistical analysis of clinical chemistry reference values. The table shows the p-value of one-way ANOVA between sex, age group categories under each sex and descriptive statistics for each clinical chemistry parameter.

Parameters	Gender (n)	One-Way ANOVA (group A vs B vs C)	One-Way ANOVA (male vs female)	Normality (Anderson-Darling)	Descriptive statistics
Alb	male (n = 345)	p value: 0.067	p value: 0.000	p value: <0.005	Mean: 40.33 (95% CI: 39.85, 40.81) SD: 4.533 Range: 28.00–54.80
	female (n = 418)	p value: 0.092		p value: <0.005	Mean: 39.11 (95% CI: 38.69, 39.51) SD: 4.229 Range: 30.00–50.70
ALP	male (n = 334)	p value: 0.447	p value: 0.000	p value: <0.005	Mean: 1.56 (95% CI: 1.52, 1.61) SD: 0.411 Range: 0.66–2.72
	female (n = 414)	p value: 0.076		p value: <0.005	Mean: 1.38 (95% CI: 1.35, 1.42) SD: 0.375 Range: 0.36–2.42
Amy	male (n = 332)	p value: 0.574	p value: 0.063	p value: <0.005	Mean: 0.86 (95% CI: 0.84, 0.88) SD: 0.254 Range: 0.31–1.59
	female (n = 408)				
D-Bil	male (n = 316)	p value: 0.310	p value: 0.000	p value: <0.005	Mean: 3.31 (95% CI: 3.17, 3.44) SD: 1.236 Range: 0.34–6.33
	female (n = 416)	p value: 0.097		p value: <0.005	Mean: 2.72 (95% CI: 2.61, 2.82) SD: 1.082 Range: 0.34–6.00
T-Bil	male (n = 319)	p value: 0.310	p value: 0.000	p value: <0.005	Mean: 14.53 (95% CI: 13.87, 15.19) SD: 5.992 Range: 5.13–30.78
	female (n = 404)	p value: 0.310		p value: <0.005	Mean: 10.84 (95% CI: 10.39, 11.30) SD: 4.646 Range: 2.74–23.26
Ca	male (n = 346)	p value: 0.508	p value: 0.001	p value: <0.005	Mean: 2.22 (95% CI: 2.19, 2.24) SD: 0.214 Range:1.58–2.7
	female (n = 427)	p value: 0.099		p value: <0.005	Mean: 2.17 (95% CI: 2.15, 2.19) SD: 0.211 Range:1.50–2.55
Chol	male (n = 330)	p value: 0.079	p value: 0.000	p value: 0.090	Mean: 4.46 (95% CI: 4.36, 4.56) SD: 0.918 Range: 2.11–6.92
	female (n = 399)	p value: 0.105		p value: <0.005	Mean: 4.07 (95% CI: 4.00, 4.15) SD: 0.802 Range: 2.16–6.50
CK	male (n = 312)	p value: 0.216	p value: 0.000	p value: <0.005	Mean: 2.67 (95% CI: 2.55, 2.79) SD: 1.053 Range: 0.44–5.95
	female (n = 403)	p value: 0.507		p value: <0.005	Mean: 1.96 (95% CI: 1.89, 2.03) SD: 0.726 Range: 0.49–4.05
Cr	male (n = 339)	p value: 0.232	p value: 0.000	p value: <0.005	Mean: 89.65 (95% CI: 88.30, 90.98) SD: 12.541 Range: 51.27–120.22
	female (n = 380)	p value: 0.172		p value: <0.005	Mean: 75.30 (95% CI: 74.50, 76.09) SD: 7.870 Range: 57.46–91.94
GGT	male (n = 296)	p value: 0.209	p value: 0.000	p value: <0.005	Mean: 0.48 (95% CI: 0.44, 0.51) SD: 0.278 Range: 0.09–1.46
	female (n = 384)	p value: 0.108		p value: <0.005	Mean: 0.31 (95% CI: 0.30, 0.33) SD: 0.147 Range: 0.09–0.85
LDH	male (n = 323)	p value: 0.107	p value: 0.710	p value: <0.005	Mean: 7.44 (95% CI: 7.34, 7.54) SD: 1.364 Range: 3.00–11.23
	Female (n = 414)	p value: 0.153			
Mg	male (n = 345)	p value: 0.067	p value: 0.056	p value: <0.005	Mean: 0.89 (95% CI: 0.87, 0.91) SD: 0.315 Range: 0.13–1.85
	female (n = 424)	p value: 0.951			
PO4	male (n = 341)	p value: 0.744	p value: 0.527	p value: 0.056	Mean: 1.00 (95% CI: 0.99, 1.02) SD: 0.175 Range: 0.49–1.49
	female (n = 413)	p value: 0.120			
SGOT	male (n = 305)	p value: 0.068	p value: 0.000	p value: <0.005	Mean: 0.53 (95% CI: 0.52, 0.55) SD: 0.134 Range: 0.17–0.88
	female (n = 392)	p value: 0.451		p value: 0.016	Mean: 0.47 (95% CI: 0.44, 0.48) SD:0.101 Range: 0.22–0.75
SGPT	male (n = 286)	p value: 0.072	p value: 0.000	p value: <0.005	Mean: 0.44 (95% CI: 0.42, 0.45) SD: 0.147 Range: 0.15–0.78
	female (n = 397)	p value: 0.071		p value: <0.005	Mean: 0.33 (95% CI: 0.32, 0.34) SD: 0.108 Range: 0.12–0.63
TGL	male (n = 323)	p value: 0.056	p value: 0.000	p value: <0.005	Mean: 1.71 (95% CI: 1.62, 1.80) SD: 0.819 Range: 0.11–4.25
	female (n = 408)	p value: 0.082		p value: <0.005	Mean: 1.36 (95% CI: 1.30, 1.42) SD: 0.652 Range: 0.10–3.15
TP	male (n = 341)	p value: 0.277	p value: 0.940	p value: <0.005	Mean: 68.96 (95% CI: 68.54, 69.34) SD: 5.950 Range:53.6–83.5
	female (n = 417)	p value: 0.228			
UA	male (n = 336)	p value: 0.538	p value: 0.000	p value: <0.005	Mean: 0.32 (95% CI: 0.32, 0.33) SD: 0.084 Range: 0.10–0.54
	female (n = 417)	p value: 0.050		p value: <0.005	Mean: 0.22 (95% CI: 0.21, 0.23) SD: 0.064 Range: 0.07–0.39
Urea	male (n = 336)	p value: 0.522	p value: 0.001	p value: <0.005	Mean: 3.63 (95% CI: 3.53, 3.74) SD: 1.001 Range: 1.18–6.33
	female (n = 413)	p value: 0.146		p value: <0.005	Mean: 3.40 (95% CI: 3.30, 3.50) SD: 1.000 Range: 0.67–6.31
Glu-r	male (n = 322)	p value: 0.223	p value: 0.009	p value: 0.016	Mean: 5.37 (95% CI: 5.28, 5.47) SD: 0.874 Range: 3.25–7.62
	female (n = 404)	p value: 0.887		p value: 0.059	Mean: 5.55 (95% CI: 5.46, 5.63) SD: 0.904 Range: 3.25–7.98
Na	male (n = 304)	p value: 0.439	p value: 0.188	p value: <0.005	Mean: 142.49 (95% CI: 142.29, 142.69) SD: 2.680 Range:135.00–148.00
	female (n = 391)	p value: 0.078			
K	male (n = 294)	p value: 0.413	p value: 0.011	p value: <0.005	Mean: 4.29 (95% CI: 4.24, 4.35) SD: 0.455 Range: 3.25–5.50
	female (n = 370)	p value: 0.114		p value: <0.005	Mean: 4.19 (95% CI: 4.14, 4.25) SD: 0.539 Range: 3.00–5.70
Cl	male (n = 308)	p value: 0.093	p value: 0.000	p value: <0.005	Mean: 105.39 (95% CI: 105.13, 105.66) SD: 2.340 Range:100.00–111.00
	female (n = 381)	p value: 0.418		p value: <0.005	Mean: 107.00 (95% CI: 106.79, 107.21) SD: 2.080 Range:102.00–112.00

Abbreviations: Alb, Albumin; ALP, Alkaline phosphatase; AMY, Amylase; Ca, Calcium; Chol, Cholesterol; CK, Creatine kinase; Cl, Chloride; Cr, Creatinine; D-Bil, Direct bilirubin; GGT, Gamma-glutamyltransferase; Glu-r, Glucose random; K, potassium; LDH, Lactate dehydrogenase; Mg, Magnesium; Na, Sodium; NHANES, National Health and Nutrition Examination Survey; PO4, Phosphorus (inorganic); SGOT, Serum glutamate oxaloacetate transaminase; SGPT, Serum glutamate pyruvate transaminase; T-Bil, Total bilirubin; TGL, Triglycerides; TP, Total protein; UA, Uric acid.

However, between sex comparison showed an insignificant difference for only six test parameters (amylase, LDH, Mg, Po4, total protein and Na). The normality testing showed that only three parameters (cholesterol, male; glucose, female; phosphorus, both sex) reference values were normally distributed. The RIs of all test parameters for both sexes established either through the parametric or non-parametric method and the 90% CI of the reference limit is shown in Table 5.

**Table 5:** The clinical chemistry RIs of healthy Bhutanese adults, 90% CI of the reference limits and other published clinical chemistry RIs for comparison.

Parameters	Gender	Present study- Bhutanese RIs (90% CI of RIs)	Indian RIs ^[^^25^^]^	IFCC–Asian RIs ^[^^25^^]^	Chinese RIs ^[^^25^^]^	Tietz Textbook RIs ^[^^26^^]^	NHANES RIs (Asia) ^[^^14^^]^
Alb (g/l)	male	32.00 (31.00–33.00)– 48.04 (47.60–48.60) ^**‡**^	39–52 ^(<45 yrs)^ 37–49 ^(≥45 yrs)^	41–51	41–52	35–52	40–51
	female	32.00 (31.00–32.00)- 47.65 (46.30–48.00) ^**‡**^	36–47	40–50			37–49
ALP (μkat/l)	male	0.89 (0.82–0.96)- 2.48 (2.41–2.62) ^**‡**^	0.68–1.89	0.66–1.63	0.85–2.11	0.90–2.18	0.65–1.79
	female	0.69 (0.60–0.80)- 2.23(2.18–2.30) ^**‡**^	-	0.54–1.43	0.66–1.63 ^(20–49 yrs)^ 0.88–2.16 ^(50–64 yrs)^	0.71–1.67	0.49–1.59
AMY (μkat/l)	male	0.45 (0.40–0.49)- 1.48 (1.41–1.50) ^**‡**^	0.61–2.29	0.77–2.23	0.49–1.57	0.46–2.23	-
	female			0.87–2.52			-
D-Bil (μmol/l)	male	1.71 (1.71–1.71)- 5.48 (5.13–6.00) ^**‡**^	-	-	-	0–3.4	-
	female	1.54 (1.34–1.71)- 5.13 (5.13–5.13) ^**‡**^	-	-	-		-
T-Bil (μmol/l)	male	5.64 (5.13–6.84)- 27.36 (27.36–30.10) ^**‡**^	6.2–23.7	-	7.1–28.8	5–21	6.8–27.4
	female	4.96 (3.76–5.13)- 22.23(21.20–22.40) ^**‡**^	4–17.3	-	6.0–22.0		5.13–20.5
Ca (mmol/l)	male	1.79 (1.70–1.83)- 2.56 (2.53–2.63) ^**‡**^	2.10–2.44	2.21–2.49	2.16–2.48	2.15–2.50	2.20–2.53
	female	1.75 (1.65–1.78) - 2.50 (2.50–2.52) ^**‡**^		2.18–2.45			2.15–2.50
Chol (mmol/l)	male	2.66 (2.52–2.80)- 6.26 (6.12–6.40) ^**†**^	3.1–6.2 ^(<45 yrs)^ 2.5–6.7 ^(≥45 yrs)^	3.5–6.7	3.19–6.16	2.93–7.15	3.05–6.71
	female	2.63 (2.60–2.70)- 5.88 (5.59–6.01) ^**‡**^	2.9–6.6	3.5–6.8	3.12–5.68 ^(20–49 yrs)^ 3.65–6.87 ^(50–64 yrs)^	3.08–7.77	3.29–7.20
CK (μkat/l)	male	1.01 (0.88–1.23)- 5.19 (4.83–5.66) ^**‡**^	0.82–5.17	0.99–4.44	1.11–4.71	0.43–2.21	0.95–17.14
	female	0.77 (0.61–0.90)- 3.53 (3.40–3.79) ^**‡**^	0.61–3.13	0.68–2.59	0.75–3.06	0.17–1.96	0.53–3.86
Cr (μmol/l)	male	60.11 (54.81–68.07)- 114.92 (114.92–114.92) ^**‡**^	58–95	61–97	57–102	62–115	60–110
	female	60.58 (59.23–61.88)- 88.40 (88.40–88.40) ^**‡**^	35–74	42–71	42–73	53–97	38–78
GGT (μkat/l)	male	0.17 (0.15–0.19)- 1.21 (1.07–1.38) ^**‡**^	0.24–1.05	0.26–1.16	0.17–1.05	0.03–0.51	0.17–1.63
	female	0.14 (0.13–0.14)- 0.68 (0.64–0.73) ^**‡**^	0.19–0.68	0.26–0.73	0.14–0.53	0.02–0.41	0.10–0.83
LDH (μkat/l)	male	5.06 (4.82–5.24) - 10.50 (10.33–10.69) ^**‡**^	1.78–3.50	2.41–4.08	2.04–3.81	1.53–5.27	1.5–3.1
	female			2.31–3.96	1.90–3.43		1.4–2.9
Mg (mmol/l)	male	0.41 (0.39–0.42) - 1.23 (1.23–1.28) ^**‡**^	0.77–1.07	-	0.78–1.0	0.66–1.07	-
	female			-			-
PO4 (mmol/l)	male/ female	0.66 (0.64–0.67)– 1.34 (1.33–1.36) ^**†**^	0.80–1.43	-	0.84–1.48	0.87–1.45	0.90–1.55
				-			0.87–1.62
SGOT (μkat/l)	male	0.31 (0.27–0.34) - 0.85 (0.83–0.87) ^**‡**^	0.34–0.90	0.28–0.60	0.27–0.66	0.14–0.34	-
	female	0.27 (0.24–0.30) - 0.68 (0.66–0.70) ^**‡**^	0.29–0.66	0.24–0.50	0.24–0.58		-
SGPT (μkat/l)	male	0.21 (0.19–0.24) - 0.76 (0.73–0.78) ^**‡**^	0.26–1.26	0.24–0.92	0.17–1.00	0.22–0.68	0.20–1.29
	female	0.17 (0.15–0.18)- 0.59 (0.55–0.61) ^**‡**^	0.17–0.63	0.19–0.53	0.10–0.58	0.17–0.48	0.17–0.79
TGL (mmol/l)	male	0.60 (0.44–0.65)- 3.80 (3.39–4.25) ^**‡**^	0.6–2.7	0.5–2.8	0.53–3.43	0.42–3.23	0.45–5.88
	female	0.44 (0.39–0.47)- 2.92 (2.78–3.04) ^**‡**^	0.5–2.1	0.4–1.7	0.46–2.28	0.44–2.62	0.40–5.23
TP (g/l)	male/ female	57.00 (56.00–57.00)- 80.00 (80.00–80.00) ^**‡**^	68–86	-	65–79	60–80	65–82
				-			64–82
UA (mmol/l)	male	0.17 (0.15–0.20)- 0.52 (0.50–0.53) ^**‡**^				0.26–0.45	0.23–0.54
	female	0.10 (0.09–0.12)- 0.37 (0.34–0.38) ^**‡**^				0.13–0.39	0.16–0.40
Urea (mmol/l)	male	1.78 (1.67–2.03)- 5.95 (5.75–6.21) ^**‡**^	2.2–6.0	2.9–7.3	3.1–7.6	2.1–7.1	-
	female	1.70 (1.50–1.86)- 5.51 (5.34–5.66) ^**‡**^	-	2.6–6.8	2.4–6.4 ^(20–49 yrs)^ 3.0–7.7 ^(50–64 yrs)^		-
Glu-r (mmol/l)	male	3.58 (3.36–3.96)- 7.41 (7.15–7.48) ^**‡**^					
	female	3.78 (3.65–3.90)- 7.32 (7.20–7.45) ^**†**^					
Na (mmol/l)	male/ female	137.00 (137.00–138.00)- 147.00 (147.00–148.00) ^**‡**^	135–146	139–146	136–144	136–145	135–143
							134–143
K (mmol/l)	male	3.44 (3.30–3.60)- 5.30 (5.20–5.40) ^**‡**^	3.8–5.0	3.7–4.7	3.7–4.7	3.5–5.1	3.4–4.7
	female	3.20 (3.10–3.30)- 5.40 (5.40–5.50) ^**‡**^					3.3–4.6
Cl (mmol/l)	male	100.73 (100.00–101.00)– 110.00 (109.00–111.00) ^**‡**^	102–113	101–108	101–109	98–107	98–108
	female	102.00 (102.00–103.00)- 111.00 (110.00–111.00) ^**‡**^					

Abbreviations: Alb, Albumin; ALP, Alkaline phosphatase; AMY, Amylase; Ca, Calcium; Chol, Cholesterol; CK, Creatine kinase; Cl, Chloride; Cr, Creatinine; D-Bil, Direct bilirubin; GGT, Gamma-glutamyltransferase; Glu-r, Glucose random; IFCC, International federation of clinical chemistry and laboratory medicine; K, potassium; LDH, Lactate dehydrogenase; Mg, Magnesium; Na, Sodium; NHANES, National Health and Nutrition Examination Survey; PO4, Phosphorus (inorganic); RIs, Reference intervals, SGOT, Serum glutamate oxaloacetate transaminase; SGPT, Serum glutamate pyruvate transaminase; T-Bil, Total bilirubin; TGL, Triglycerides; TP, Total protein; UA, Uric acid. Symbols

‡, Non-parametric percentile estimate

†, Parametric percentile estimate.

### Haematology RIs

The age range of individuals constituting the haematology reference sample group for both sexes was 18–60 years old. Within the same sex, there is no significant difference between the age group categories for all test parameters (Table 6).

**Table 6:** The statistical analysis of haematology reference values. The table shows the p-value of one-way ANOVA between sex, age group categories under each sex and descriptive statistics for each haematology parameter.

Parameters	Gender (n)	One-Way ANOVA (group A vs B vs C)	One-Way ANOVA (male vs female)	Normality (Anderson-Darling)	Descriptive statistics
TLC	male (n = 350)	p value: 0.585	p value: 0.009	p value: 0.110	Mean: 6.59 (95% CI: 6.43, 6.75) SD: 1.517 Range: 3.00–11.00
	female (n = 427)	p value: 0.445		p value: 0.009	Mean: 6.87 (95% CI: 6.73, 7.02) SD: 1.547 Range: 3.37–11.09
Neut (%)	male (n = 351)	p value: 0.084	p value: 0.635	p value: 0.118	Mean: 56.18 (95% CI: 55.58, 56.79) SD: 8.596 Range: 31.40–78.30
	female (n = 427)	p value: 0.056			
Lymph (%)	male (n = 351)	p value: 0.071	p value: 0.064	p value: 0.565	Mean: 33.05 (95% CI: 32.53, 33.57) SD: 7.383 Range:12.70–52.50
	female (n = 425)	p value: 0.265			
Mono (%)	male (n = 346)	p value: 0.376	p value: 0.000	p value: 0.099	Mean: 7.25 (95% CI: 7.05, 7.45) SD: 1.896 Range: 2.60–12.30
	female (n = 428)	p value: 0.211		p value: 0.027	Mean: 6.56 (95% CI: 6.38, 6.74) SD: 1.891 Range: 1.00–12.00
Eosi (%)	male (n = 335)	p value: 0.404	p value: 0.092	p value: <0.005	Mean: 2.81 (95% CI: 2.68, 2.94) SD: 1.818 Range: 0.00–8.10
	female (n = 412)	p value: 0.572			
Baso (%)	male (n = 339)	p value: 0.637	p value: 0.015	p value: <0.005	Mean: 0.46 (95% CI: 0.42, 0.49) SD: 0.345 Range: 0.00–1.40
	female (n = 425)	p value: 0.986		p value: <0.005	Mean: 0.40 (95% CI: 0.37, 0.43) SD: 0.324 Range: 0.00–1.30
Neut #	male (n = 349)	p value: 0.305	p value: 0.088	p value: <0.005	Mean: 3.80 (95% CI: 3.72, 3.88) SD: 1.162 Range:1.45–7.17
	female (n = 425)	p value: 0.229			
Lymph #	male (n = 345)	p value: 0.133	p value: 0.000	p value: <0.005	Mean: 2.09 (95% CI: 2.03, 2.15) SD: 0.556 Range: 0.77–3.49
	female (n = 427)	p value: 0.291		p value: <0.005	Mean: 2.29 (95% CI: 2.24, 2.35) SD: 0.591 Range: 0.91–3.84
Mono #	male (n = 338)	p value: 0.643	p value: 0.191	p value: <0.005	Mean: 0.45 (95% CI: 0.44, 0.46) SD: 0.149 Range: 0.04–0.86
	female (n = 425)	p value: 0.317			
Eosi #	male (n = 335)	p value: 0.287	p value: 0.589	p value: <0.005	Mean: 0.19 (95% CI: 0.18, 0.20) SD: 0.128 Range:0.00–0.58
	female (n = 411)	p value: 0.425			
Baso #	male (n = 348)	p value: 0.385	p value: 0.002	p value: <0.005	Mean: 0.03 (95% CI: 0.03, 0.03) SD: 0.026 Range: 0.00–0.11
	female (n = 417)	p value: 0.782		p value: <0.005	Mean: 0.03 (95% CI: 0.02, 0.03) SD: 0.021 Range:0.00–0.08
TRBC	male (n = 351)	p value: 0.165	p value: 0.000	p value: 0.506	Mean: 5.35 (95% CI: 5.30, 5.40) SD: 0.434 Range: 4.2–6.54
	female (n = 430)	p value: 0.113		p value: 0.112	Mean: 4.77 (95% CI: 4.73, 4.81) SD: 0.414 Range: 3.60–5.94
Hb	male (n = 352)	p value: 0.065	p value: 0.000	p value: 0.559	Mean: 155.58 (95% CI: 154.19, 156.98) SD: 13.300 Range: 117.00–189.00
	female (n = 430)	p value: 0.600		p value: <0.005	Mean: 136.14 (95% CI: 134.82, 137.45) SD: 13.860 Range: 101.00–168.00
Hct	male (n = 349)	p value: 0.052	p value: 0.000	p value: 0.342	Mean: 0.46 (95% CI: 0.46, 0.47) SD: 0.033 Range: 0.37–0.55
	female (n = 429)	p value: 0.078		p value: 0.020	Mean: 0.41 (95% CI: 0.41, 0.42) SD: 0.034 Range: 0.32–0.49
MCV	male (n = 341)	p value: 0.516	p value: 0.035	p value: 0.521	Mean: 86.99 (95% CI: 86.51, 87.47) SD: 4.485 Range: 74.20–98.20
	female (n = 415)	p value: 0.081		p value: <0.005	Mean: 87.70 (95% CI: 87.25, 88.16) SD: 4.734 Range: 73.10–100.00
MCH	male (n = 343)	p value: 0.970	p value: 0.002	p value: 0.482	Mean: 29.30 (95% CI: 29.07, 29.53) SD: 2.121 Range: 23.80–34.50
	female (n = 421)	p value: 0.116		p value: <0.005	Mean: 28.77 (95% CI: 28.54, 29.00) SD: 2.404 Range: 22.50–34.40
MCHC	male (n = 347)	p value: 0.703	p value: 0.000	p value: 0.011	Mean: 336.45 (95% CI: 335.04, 337.87) SD: 13.410 Range:303.00–370.00
	female (n = 433)	p value: 0.130		p value: <0.005	Mean: 329.59 (95% CI: 328.16, 331.02) SD: 15.140 Range: 291.00–368.00
RDW-CV	male (n = 294)	p value: 0.438	p value: 0.054	p value: <0.005	Mean: 13.07 (95% CI: 13.01, 13.13) SD: 0.790 Range:11.10–15.60
	female (n = 351)	p value: 0.742			
RDW-SD	male (n = 299)	p value: 0.173	p value: 0.029	p value: <0.005	Mean: 40.30 (95% CI: 40.04, 40.56) SD: 2.306 Range: 34.90–46.50
	female (n = 355)	p value: 0.231		p value: 0.033	Mean: 40.69 (95% CI: 40.46, 40.93) SD: 2.267 Range: 34.60–46.60
Platelet	male (n = 349)	p value: 0.372	p value: 0.000	p value: 0.486	Mean: 249.16 (95% CI: 243.22, 255.11) SD: 56.500 Range: 97.00–404.00
	female (n = 425)	p value: 0.131		p value: 0.042	Mean: 266.48 (95% CI: 260.56, 272.40) SD: 62.070 Range:117.00–427.00
PDW	male (n = 277)	p value: 0.866	p value: 0.302	p value: <0.005	Mean: 12.48 (95% CI: 12.29, 12.67) SD: 2.423 Range: 6.80–18.70
	female (n = 355)	p value: 0.823			
MPV	male (n = 343)	p value: 0.843	p value: 0.007	p value: 0.477	Mean: 10.65 (95% CI: 10.53, 10.77) SD: 1.149 Range: 7.60–13.40
	female (n = 429)	p value: 0.609		p value: 0.260	Mean: 10.88 (95% CI: 10.77, 11.00) SD: 1.230 Range:7.60–14.00
PCT	male (n = 282)	p value: 0.482	p value: 0.000	p value: 0.067	Mean: 0.26 (95% CI: 0.25, 0.27) SD: 0.064 Range: 0.09–0.44
	female (n = 360)	p value: 0.129		p value: 0.050	Mean: 0.29 (95% CI: 0.28, 0.30) SD: 0.069 Range: 0.12–0.46
P-LCR	male (n = 238)	p value: 0.812	p value: 0.011	p value: 0.022	Mean: 32.04 (95% CI: 31.06, 33.01) SD: 7.644 Range: 16.30–51.20
	female (n = 304)	p value: 0.893		p value: <0.005	Mean: 33.85 (95% CI: 32.88, 34.82) SD: 8.600 Range: 13.70–55.90
PT	male (n = 341)	p value: 0.052	p value: 0.660	p value: <0.005	Mean: 12.84 (95% CI: 12.76, 12.91) SD: 1.074 Range: 10.70–15.90
	female (n = 420)	p value: 0.150			

Abbreviation: Baso, Basophil; Eosi, Eosinophil; Hb, Haemoglobin; Hct, Haematocrit; Lymph, Lymphocyte; MCH, Mean corpuscular haemoglobin; MCHC, Mean corpuscular haemoglobin concentration; MCV, Mean corpuscular volume; Mono, Monocyte; MPV, Mean platelet volume; Neut, Neutrophil; NHANES, National health and nutrition examination survey; PCT, Plateletcrit; PDW, Platelet distribution width; P-LCR, Platelet-large cell ratio; PT, Prothrombin time; RDW-SD, Red cell distribution width- standard deviation; RDW-CV, Red cell distribution width- coefficient of variation; TLC, Total leukocyte count; TRBC, Total red blood cell count; Symbols: #, Absolute count; %, Percentage.

However, between sex comparison showed an insignificant difference for only nine test parameters (neutrophil%, lymphocyte%, eosinophil%, neutrophil abs, monocyte abs, eosinophil abs, RDW-CV, PDW and PT). The normality testing showed that 36.59% of reference values were normally distributed. The RIs of all test parameters for both sexes established either through the parametric or non-parametric method and the 90% CI of the reference limit is shown in Table 7.

**Table 7:** The haematology RIs of healthy Bhutanese adults, 90% CI of the reference limits and other published haematology RIs for comparison.

Parameters	Gender	Present study- Bhutanese RIs (90% CI of RIs)	Chinese RIs ^[^^34^^]^	American RIs ^[^^34^^]^	Malaysian RIs ^[^^34^^]^	Dacie and Lewis RIs ^[^^37^^]^	NHANES (Asia) RIs ^[^^14^^]^
TLC (x10^9^/l)	male	3.62 (3.39–3.84)- 9.56 (9.34–9.80) ^**†**^	3.64–9.39	4.5–11.0	3.8–9.7	4.0–10.0	3.8–11.7
	female	4.21 (4.01–4.31)- 10.29 (9.98–10.54) ^**‡**^			3.4–10.1		3.9–10.8
Neut (%)	male/ female	39.33 (38.47–40.20)- 73.03 (72.16–73.89) ^**†**^	41.5–73.8	40–70	42.8–69.2	40–80	40.2–75.4
					43.2–70.6		39.8–75.0
Lymph (%)	male/ female	18.58 (17.83–19.32)- 47.52 (46.78–48.27) ^**†**^	18.6–48.7	22–44	18.5–47.7	20–40	16.5–48.8
					19.2–47.5		16.7–49.6
Mono (%)	male	3.53 (3.25–3.82)- 10.97 (10.68–11.25) ^**†**^	3.2–9.5	4–11	-	2–10	3.8–11.1
	female	3.00 (2.39–3.08)- 10.43 (10.00–10.70) ^**‡**^			-		3.3–10.6
Eosi (%)	male/ female	0.40 (0.30–0.60)- 7.40 (7.10–7.60) ^**‡**^	0.4–8.1	0–8	-	1–6	0.7–8.9
					-		0.6–8.3
Baso (%)	male	0.00 (0.00–0.00)- 1.20 (1.10–1.30) ^**‡**^	0.1–1.1	0–3	-	<1–2%	0.0–2.0
	female	0.00 (0.00–0.00)- 1.10 (1.00–1.10) ^**‡**^			-		0.1–1.8
Neut # (x10^9^/l)	male/ female	1.96 (1.81–2.06)- 6.50 (6.21–6.64) ^**‡**^	1.80–6.30	1.8–7.7	1.58–5.94	2.0–7.0	-
					1.55–6.07		-
Lymph # (x10^9^/l)	male	1.00 (1.00–1.26)- 3.29 (3.11–3.44) ^**‡**^	1.06–3.2	1.0–4.8	1.14–3.22	1.0–3.0	-
	female	1.22 (1.08–1.37)- 3.62 (3.52–3.71) ^**‡**^			1.05–3.29		-
Mono # (x10^9^/l)	male/ female	0.19 (0.15–0.20)- 0.77 (0.75–0.80) ^**‡**^	0.16–0.62	0–0.8	0.15–0.67	0.2–1.0	-
					0.1–0.74		-
Eosi # (x10^9^/l)	male/ female	0.03 (0.02–0.03)- 0.52 (0.50–0.54) ^**‡**^	0.02–0.52	0–0.045	0.08–0.28	0.02–0.5	-
					0.03–0.27		-
Baso # (x10^9^/l)	male	0.00 (0.00–0.00)- 0.10 (0.09–0.10) ^**‡**^	0.00–0.06	0–0.2	0.01–0.05	0.02–0.1	-
	female	0.00 (0.00–0.00)- 0.07 (0.07–0.08) ^**‡**^			0.01–0.05		-
TRBC (x10^12^/l)	male	4.50 (4.43–4.56)- 6.20 (6.14–6.27) ^**†**^	4.28–5.81	4.5–5.9	4.18–6.06	4.5–5.5	4.06–5.97
	female	3.96 (3.90–4.01)- 5.58 (5.53–5.64) ^**†**^	3.81–5.13	4.0–5.2	3.52–5.16	3.8–4.8	3.66–5.05
Hb (g/l)	male	129.51 (127.52–131.50)- 181.65 (179.66–183.64) ^**†**^	133–175	135–175	120–165	130–170	122–169
	female	106.00 (103.00–107.00)- 160.00 (158.00–163.00) ^**‡**^	115–152	120–160	98–138	120–150	105–149
Hct (l/l)	male	0.40 (0.39–0.40)- 0.52 (0.52–0.53) ^**†**^	0.40–0.51	0.41–0.53	0.375–0.498	0.4–0.5	0.37–0.49
	female	0.34 (0.34–0.35)- 0.48 (0.47–0.48) ^**‡**^	0.35–0.46	0.36–0.46	0.318–0.424	0.36–0.46	0.32–0.44
MCV (fl)	male	78.20 (77.52–78.88)- 95.78 (95.10–96.46) ^**†**^	82.3–99.2	80–100	78.9–95.7	83–101	69.9–99.8
	female	76.04 (74.00–78.20)- 96.00 (95.00–98.10) ^**‡**^			77.5–94.5		67.8–97.8
MCH (pg)	male	25.14 (24.82–25.46)- 33.46 (33.14–33.78) ^**†**^	27.0–33.7	-	25.4–31.1	27–32	22.3–34.0
	female	23.10 (23.00–23.90)- 33.10 (32.80–33.40) ^**‡**^		-	24.8–31.2		22.1–33.8
MCHC (g/l)	male	309.00 (307.00–314.00)- 364.30 (362.00–368.00) ^**‡**^	316–354	-	306–348	315–345	318–360
	female	303.85 (300.00–305.00)- 361.15 (358.00–362.00) ^**‡**^		-	294–644		323–358
RDW-CV (%)	male/ female	11.80 (11.70–11.90)- 14.90 (14.80–15.00) ^**‡**^	12.0–13.6*	-	-	11.6–14	11.4–14.6
			12.1–14.3*	-	-		11.3–15.7
RDW-SD (fl)	male	36.10 (35.30–36.50)- 45.50 (44.90–46.40) ^**‡**^	37.1–45.7*	-	-	39–46	-
	female	36.38 (35.70–36.80)- 45.90 (45.10–46.10) ^**‡**^	38.2–49.2*	-	-		-
Platelet (x10^9^/l)	male	138.42 (129.92–146.92)- 359.90 (351.40–368.40) ^**†**^	127–350	150–350	167–376	150–410	152–325
	female	151.30 (134.00–161.00)- 399.35 (384.00–410.00) ^**‡**^			158–410		139–370
PDW (fl)	male/ female	8.10 (7.90–8.50)- 17.90 (17.30–18.10) ^**‡**^	10.1–16.1*	-	-	-	-
			9.9–15.4*	-	-	-	-
MPV (fl)	male	8.40 (8.22–8.57)- 12.90 (12.73–13.08) ^**†**^	9.3–12.1*	-	-	-	6.6–10
	female	8.50 (8.30–8.64)- 13.29 (13.12–13.46) ^**†**^	9.1–11.9*	-	-	-	6.8–10
PCT (%)	male	0.13 (0.12–0.15)- 0.39 (0.37–0.40) ^**†**^	0.17–0.32*	-	-	-	-
	female	0.15 (0.14–0.16)- 0.43 (0.42–0.44) ^**†**^	0.18–0.39*	-	-	-	-
P-LCR (%)	male	18.20 (16.90–20.00)- 47.42 (46.70–49.80) ^**‡**^	18.5–42.3*	-	-	-	-
	female	19.46 (18.00–20.80)- 54.05 (50.60–54.90) ^**‡**^	17.5–42.3*	-	-	-	-
PT (second)	male/ female	11.00 (11.00–11.02)- 15.10 (15.00–15.20) ^**‡**^	-	-	-		-
			-	-	-		-

Abbreviation: Baso, Basophil; Eosi, Eosinophil; Hb, Haemoglobin; Hct, Haematocrit; Lymph, Lymphocyte; MCH, Mean corpuscular haemoglobin; MCHC, Mean corpuscular haemoglobin concentration; MCV, Mean corpuscular volume; Mono, Monocyte; MPV, Mean platelet volume; Neut, Neutrophil; NHANES, National health and nutrition examination survey; PCT, Plateletcrit; PDW, Platelet distribution width; P-LCR, Platelet-large cell ratio; PT, Prothrombin time; RDW-SD, Red cell distribution width- standard deviation; RDW-CV, Red cell distribution width- coefficient of variation; TLC, Total leukocyte count; TRBC, Total red blood cell count; Symbols

‡, Non-parametric percentile estimate

†, Parametric percentile estimate

*, Reference intervals from reference no 36

#, Absolute count; %, Percentage.

## Discussion

There is no literature on RIs of the Bhutanese population for clinical laboratory test parameters. The clinical laboratories in Bhutan have been using RIs from the standard textbooks without even verifying their applicability. Since the majority of the textbook RIs are established using western populations and clinical laboratories different from our setup, the use of textbook RIs is not appropriate. In this regard, the establishment of clinical chemistry and haematology RIs specific to the Bhutanese population has the potential to improve the quality of healthcare services in Bhutan. Initially, the international organizations recommended clinical laboratories establish their RIs; however, the recent literature on RIs recommends the use of common RIs if the population served is indifferent and the quality between the two laboratories is similar [5, 23, 24]. Since Bhutan is one of the least populated countries (∼19 persons per km^2^) [16] with the entire population falling under the broad classification of Asian descent, a common RIs for all Bhutanese ethnicities is suitable. Furthermore, common RIs for all clinical laboratories in Bhutan are appropriate because nearly all clinical laboratories have similar equipment, reagents, environmental conditions, and quality system as they are owned by the government. The most common way to partition clinical laboratory data is by age and sex and further adulthood can be divided into decades [6]. Following this trend, initially, we partitioned the reference sample group into male and female followed by a 13-years age grouping under each sex. In our study, there is no significant difference between sex for six clinical chemistry analytes (Amy, LD, Mg, Po4 and total protein). Similar findings of indifference between sex for these parameters are reported by several other studies [14, 25, 26]. Although these studies have reported significant differences among age group categories for several parameters, we observed no significant difference within age group categories for all test parameters in the Bhutanese population. The biological reason for this different observation remains unknown, but methodological differences in the age grouping between studies can contribute to this difference. Our RIs established for 23 Clinical chemistry parameters were comparable to various other published RIs [13, 14, 25, 27, 28]; however, the Bhutanese LDH RI is substantially higher on both limits compared to the RIs published for Asian descendent. The Bhutanese LDH RI is more comparable to the RI established in the African population [28–30]. Seventy-nine per cent of the Bhutanese population is involved in agriculture and livestock farming [31] and therefore subjected to constant physical strain and stress. Studies on the impact of exercise on Clinical chemistry parameters have reported that exercise significantly increases the serum LDH level [32, 33]. Consistent with these reports, the physically demanding life of the Bhutanese may be responsible for a higher level of serum LDH.

For haematology, we observed an insignificant difference between sex for 9 test parameters (nine CBC parameters and PT). The haematological difference between sexes was found to be variable in various populations [14, 34–37]. This variable between sexes may be due to the actual biological differences or differences in study design. In our study, many red cell series test parameters were found to be higher in males than females. Similar findings have been reported in other published literature. The higher RBC count and Hb level in males are suggested to be due to an effect of androgen and estrogen on erythropoiesis [35, 36, 38, 39]. The lower limit of our Hb level is lower than the limit (≥120 g/l) specified by the WHO for non-pregnant women [40]. The WHO has defined this limit in 1968 based on a few studies conducted on the European and American populations, yet this cut-off has been applied to all other populations across the globe [41]. Sex, ethnicity and physiological status are observed to influence the normal Hb level; therefore, new lower limits for Hb based on sex, ethnicity and age have been proposed [42]. Like our findings, the National Health and Nutrition Examination Survey (NHANES) and other RI studies from Malaysia and Africa have reported lower limits lower than the WHO limit [14, 34]. Consistent with various published literature [43–46], we also observed a higher platelet count in females than in males. This difference is suggested to be due to the different hormonal profiles or compensation mechanisms associated with menstrual blood loss [47]. The platelet RI study conducted on the UK population by the Ali U et al reported that there is a significant difference in platelet indices RI between sexes [48]. Consistent with this finding, we also observed insignificant differences only for PDW; however, variable observations between sexes were also published [45, 49]. The leukocyte counts are reported to be higher in females than males and they reverse in old age [46, 50]. In agreement with this published literature, we observed a significantly higher total white cell count in females than males in healthy Bhutanese adults.

## Conclusions

The RIs established through this study are applicable to all the Bhutanese population (adult) since representative samples from all the major ethnic groups have been obtained and all Bhutanese ethnic groups fall under the broad classification of Asian descent. The clinical laboratories however need to validate the transference of these RIs following the recommended procedures before using them for clinical purposes. Since the present RIs are based on healthy Bhutanese adults for routine clinical chemistry and haematological test parameters, RIs for specialised tests and other age groups must be established.

### Limitation of the study

The study combined all the participants from diverse ethnic groups into one category to establish a common RI. Although all ethnic groups in Bhutan fall under the broad category of Asian descent, it is worthwhile to explore physiological differences among the ethnic groups in future studies.
